# Changes in Sales of Tobacco and Nicotine Replacement Therapy Products Before and During the COVID-19 Pandemic

**DOI:** 10.5888/pcd20.220406

**Published:** 2023-08-17

**Authors:** Jaesang Sung, Sundar S. Shrestha, Yoonsang Kim, Sherry Emery, Xu Wang

**Affiliations:** 1 Office on Smoking and Health, National Center for Chronic Disease Prevention and Health Promotion, Centers for Disease Control and Prevention, Atlanta, Georgia; 2 NORC at the University of Chicago, Chicago, Illinois

## Abstract

**Introduction:**

The COVID-19 pandemic and its associated social distancing policies such as lockdowns and quarantine influenced people’s lives and health behaviors. We comprehensively assessed national trends in sales of cigarettes, cigars, e-cigarettes, and over-the-counter nicotine replacement therapy (NRT) products before and during the pandemic, allowing for cross-product comparisons. Stockpiling behavior was also assessed.

**Methods:**

We used US national tobacco and over-the-counter NRT retail store scanner data (excluding internet, specialty/vape store, and prescription sales) collected at 4-week intervals by NielsenIQ from December 2018 to June 2021. We applied an interrupted time-series model to assess differences in tobacco product and NRT unit sales before and during the pandemic. We defined the prepandemic period as December 16, 2018, through April 4, 2020, pandemic as starting on April 5, 2020, through June 26, 2021, and the stockpiling period as one 4-week period before the pandemic started.

**Results:**

Four-week cigarette, e-cigarette, and cigar unit sales on average increased by 11.5% (*P* = .006), 37.1% (*P* < .001), and 26.1% (*P* < .001) respectively, while 4-week NRT unit sales decreased on average by 13.1% (*P* < .001), during the pandemic compared with the prepandemic period. Stockpiling was associated with increases in sales of all tobacco products and NRT products.

**Conclusion:**

Unit sales of assessed tobacco products increased while NRT unit sales decreased during the COVID-19 pandemic, compared with the prepandemic period. These changes may suggest an increase in the intensity of tobacco product use or stockpiling of tobacco products among people who use tobacco.

**Summary**

**What is already known on this topic?**

Cigarette smoking is associated with severe COVID-19 illness. Research shows mixed evidence about changes in the intensity of tobacco product use during the pandemic.

**What is added by this report?**

We assessed trends in sales of cigarettes, cigars, e-cigarettes, and nicotine replacement therapy (NRT) products before and during the COVID-19 pandemic, as well as possible stockpiling of these products. We made cross-product comparisons of changes in sales in the short term (ie, stockpiling period) and the longer term.

**What are the implications for public health practice?**

Increases in tobacco product sales during the COVID-19 pandemic were a public health concern. Decreases in NRT sales during the pandemic may suggest a drop in use of evidence-based cessation supports or quit attempts generally.

## Introduction

In March 2020, the World Health Organization declared disease caused by the SARS-CoV-2 virus (COVID-19) to be a global pandemic. As of November 20, 2022, more than 98.1 million cases and more than 1 million COVID-19 deaths had been reported in the US (1).

Cigarette smoking has been associated with severe COVID-19 illness (2). The perceived threat of adverse health outcomes associated with smoking when contracting COVID-19 might motivate people who smoke to quit (3). Yet stress due to fear of the pandemic and the feeling of isolation due to mobility restrictions might increase cigarette and e-cigarette use among current users or cause relapse among people who had previously quit (3,4). Research shows mixed evidence about changes in the intensity of tobacco product use during the pandemic (4–7).

Purchasing behavior may have also changed during the COVID-19 pandemic. Recent literature estimated initial increased sales for cigars (8) and cigarettes (9) but decreased sales for nicotine replacement therapy (NRT) products (10) during the pandemic, compared with the prepandemic period. Additionally, people who use tobacco might have stockpiled tobacco products in anticipation of or in response to the COVID-19 lockdown (11). Stockpiling and bulk purchasing behaviors have been common among consumers during the pandemic (8,12). The presumed over-purchase of tobacco products in the short term might lead to long-term increases in purchases even after the pandemic ends (13).

Using NielsenIQ sales data, this study complements previous studies that assessed changes in tobacco use and sales (3,4,8–10,14). However, unlike previous studies that assessed changes in sales of individual products such as cigars (8), cigarettes (9), and NRT products (10), this study examined the nationwide sales trends of cigarettes, e-cigarette, cigars, and over-the-counter (OTC) NRT products all at once, providing cross-product comparisons in their predicted sales trends before and during the pandemic. In addition, this study assessed possible stockpiling of all these tobacco products and OTC NRT products, allowing assessment of changes in sales in the short term (ie, stockpiling period) and in longer-term sales since the pandemic began.

## Methods

We used US retail store scanner data from NielsenIQ for sales of tobacco products (cigarettes, e-cigarettes, and cigars) and OTC NRT products (nicotine gum, patches, and lozenges). The data represent sales in the 48 contiguous states and the District of Columbia and do not include Hawaii and Alaska. NielsenIQ data provided unit sales for each UPC (Universal Product Code) in 4-week aggregates from the period ending January 12, 2019, through the period ending June 26, 2021 (33 four-week periods).

NielsenIQ data use a proprietary sample-based method to calculate representative sales for retail stores, using both in-store scanning tools and in-person audits. The data represent sales in convenience stores (chain, franchise, and independent stores with and without provisions for gasoline) and sales in the “All Outlets Combined” channel, which represents sales in food and grocery stores, pharmacies, mass merchandisers, warehouse club stores, discount and dollar stores, and US military commissaries. Retail sales data are estimated to cover 77% of retail stores in the US (15). Internet sales and tobacco or vape shop sales are not included.

### Measures

In this study, “tobacco” refers to commercial tobacco products, not to tobacco used for medicinal and spiritual purposes by some American Indian communities. The total unit sales were calculated for every 4 weeks during the study period. One unit sale of cigarettes equals 1 pack of 20 cigarettes. E-cigarettes were classified as disposable e-cigarettes (single-use products that cannot be recharged or refilled), prefilled pods or cartridges (pods or cartridges for refill and items like starter kits that contain a rechargeable device and pods or cartridges), and e-liquid bottles for refill. One unit sale of an e-cigarette equals 1 disposable e-cigarette, 1 e-liquid bottle, or 5 prefilled pods or cartridges. A small fraction of products could not be categorized due to insufficient information in the NielsonIQ data and via online search. We excluded sales of rechargeable devices only (ie, without pods or cartridges included), accessories, and uncategorized products. Cigars were classified as large cigars, little cigars, and cigarillos. One unit sale of cigars equals 1 large cigar, 1 pack of 20 little cigars, or 1 pack of 2 cigarillos. We excluded uncategorized cigar products from the analysis. Lastly, OTC NRT products approved by the US Food and Drug Administration (FDA) included nicotine patches (7 mg, 14 mg, or 21 mg nicotine strength), nicotine lozenges (2 mg or 4 mg nicotine strength), and nicotine gum (2 mg or 4 mg nicotine strength). One unit equals 1 patch with 7 mg nicotine strength, 1 lozenge with 2 mg nicotine strength, or 1 piece of gum with 2 mg nicotine strength. We excluded NRT products with unusual nicotine strength.

### Statistical analysis

We used an interrupted time series model (16) to assess the association of changes in tobacco product and NRT product sales with the COVID-19 pandemic. For each product, we estimated a linear and polynomial regression of monthly unit sales in logarithmic scale, with a binary indicator specified as 1 for April 5, 2020, through June 26, 2021, to define the pandemic period and 0 for the period before the pandemic. In response to the pandemic, many state and local governments instituted restrictions on nonessential businesses and issued stay-at-home orders, starting on March 19, 2020, in California (17). We defined a hypothetical stockpiling period in anticipation of or in response to pandemic restrictions as a binary indicator of 1 for the 4-week period from March 8 to April 4, 2020, and 0 otherwise. To reflect the nonlinear trends of sales in the interrupted time series model, the polynomials terms (ie, 1, 2, and 3) were used and determined independently for each tobacco product type by assessing residuals and model-fit statistics. We exponentiated the estimated rate of the 4-week change in unit sales to derive the monthly percentage changes (MPCs) in unit sales during the study period. We also used the models to estimate the effects of the pandemic and potential stockpiling behaviors on unit sales. To calculate the estimated percentage change in unit sales, we exponentiated each regression coefficient for the binary indicators for the pandemic period and stockpiling month, and then subtracted 1. Significant change was indicated by a *P* value of ≤.05.

Two national tobacco-related events also occurred during the study period — the passage of national legislation to increase the legal tobacco purchase age to 21 years (Tobacco 21) on December 20, 2019 (18), and FDA’s flavored e-cigarette enforcement guidance effective February 6, 2020 (19). These events could have influenced the estimated associations of tobacco product and NRT product sales with the pandemic and stockpiling behaviors. Therefore, as a sensitivity check, in the models we controlled for 2 binary variables representing the periods that each event was in effect: 1) a binary indicator for the period that Tobacco 21 was enacted, that is, from the 4-week period ending January 11, 2020, to June 26, 2021, and 2) a binary indicator for the period that FDA’s regulation on flavored e-cigarette sales was in effect, that is, from the 4-week period ending March 7, 2020, to June 26, 2021.

Because error terms are typically correlated in time series data, we measured the autocorrelation function in the model; these autocorrelation function values were within 95% CIs and tapered quickly. We used the Prais–Winsten method to estimate model parameters considering serial correlation of the errors as a sensitivity analysis (20). We used Stata version 16 (StataCorp LLC) to conduct all analyses.

## Results

### Cigarette sales

The average 4-week unit sales of cigarettes was 701.2 million units before the pandemic and 692.0 million units during the pandemic. Cigarette unit sales increased through the period ending June 29, 2019, peaking at more than 748.5 million units, and then declined until early 2020 (Figure). Cigarette unit sales jumped by about 28.8 million units during the 4-week period ending April 4, 2020 (model-based MPC = 8.5%; *P* = .008), and then further increased to levels higher than prepandemic sales, at 765.0 million units in the period ending June 27, 2020, before declining again through the period ending March 6, 2021, and increasing again afterwards.

**Figure:**
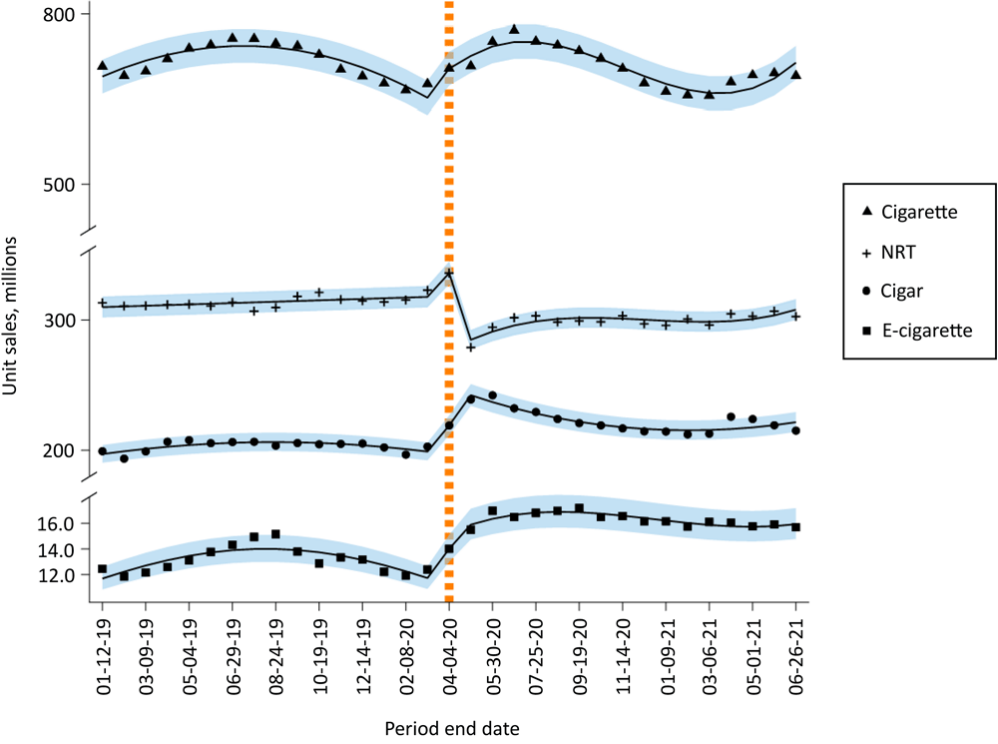
Unit sales of cigarette, e-cigarette, cigar, and over-the-counter nicotine replacement therapy (NRT) products, January 2019–June 2021. NRT products are gum, patches, and lozenges. Vertical dashed line indicates beginning of COVID-19 period (April 5, 2020–June 26, 2021). Solid lines indicate estimated unit sales based on the interrupted time-series model; shaded areas indicate 95% CIs. Different models were found to be the best fit for each product.

**Table:** 

Period end date	Type of product, sales in millions of units			
	Cigarette	E-cigarette	Cigar	NRT
2019–01-12	692.4	12.5	199.3	313.2
2019–02-09	674.5	11.9	193.6	310.7
2019–03-09	683.1	12.2	199.2	310.9
2019–04-06	707.3	12.6	206.6	311.8
2019–05-04	728.1	13.1	207.8	312.0
2019–06-01	735.2	13.8	205.5	310.7
2019–06-29	748.5	14.3	206.4	313.5
2019–07-27	748.2	14.9	206.6	306.7
2019–08-24	738.2	15.2	203.6	309.5
2019–09-21	733.0	13.8	205.6	318.1
2019–10-19	715.6	12.9	204.6	321.2
2019–11-16	686.6	13.3	204.9	315.7
2019–12-14	673.7	13.2	205.2	314.8
2020–01-11	660.5	12.2	202.2	313.8
2020–02-08	648.1	11.9	196.7	315.4
2020–03-07	659.5	12.4	202.9	322.8
2020–04-04	688.3	14.0	219.1	336.0
2020–05-02	693.1	15.5	239.0	278.9
2020–05-30	741.6	17.0	242.2	294.4
2020–06-27	765.0	16.5	232.3	301.8
2020–07-25	742.5	16.8	229.4	303.1
2020–08-22	734.5	17.0	224.1	298.4
2020–09-19	723.1	17.2	220.9	299.3
2020–10-17	707.8	16.5	219.2	298.5
2020–11-14	688.2	16.6	216.8	303.2
2020–12-12	661.0	16.2	214.6	297.0
2021–01-09	645.5	16.2	214.4	295.8
2021–02-06	639.1	15.8	212.2	300.7
2021–03-06	638.2	16.1	212.8	296.1
2021–04-03	662.9	16.1	225.8	304.7
2021–05-01	675.8	15.8	224.0	302.9
2021–05-29	679.7	15.9	219.3	306.7
2021–06-26	674.6	15.7	215.1	302.7

The 4-week cigarette unit sales increased on average by 11.5% (*P* = .006) during the pandemic compared with the prepandemic period (Table 1). The stockpiling period was associated with 12.5% (*P* < .001) greater unit sales, on average, compared with all other 4-week unit sales during the study period.

**Table 1:** Estimated Association of US Unit Sales of Tobacco Products and Nicotine Replacement Therapy (NRT) Products With the COVID-19 Pandemic and Potential Stockpiling Periods, January 2019–June 2021^a^

Product	Estimated association with COVID-19 pandemic^b^		Estimated association with the period of potential stockpiling^c^	
	Estimate effect, % (95% CI)	*P* value^d^	Estimate effect, % (95% CI)	*P* value^d^
Cigarettes	11.5 (3.5 to 20.2)	.006	12.5 (5.5 to 19.8)	<.001
E-cigarettes	37.1 (21.7 to 54.5)	<.001	25.6 (13.5 to 39.0)	<.001
Cigars	26.1 (20.9 to 31.6)	<.001	11.3 (6.2 to 16.6)	<.001
NRT products^e^	−13.1 (−16.2 to −9.8)	<.001	5.6 (2.5 to 8.8)	<.001

a For cigarette and e-cigarette unit sales, linear and quadratic terms were included in the periods before the pandemic and another set of linear, quadratic, and cubic terms were added for the period during the pandemic. For cigar unit sales, linear and quadratic terms were included before and during the pandemic. For NRT unit sales, a linear term was added before the pandemic, and a set of linear, quadratic, and cubic terms were included during the pandemic.

b The pandemic was defined as beginning of the 4-week period starting on April 5, 2020, through June 26, 2021. The prepandemic period was defined as the 4-week period before April 5, 2020.

c The stockpiling period was defined as the 4-week periods from March 8, 2020, through April 4, 2020.

d Determined by *t* tests; *P* ≤ .05 considered significant.

e NRT products sold over the counter; includes gum, patches, and lozenges.

### E-cigarette sales

The average 4-week unit sales of e-cigarettes were 13.2 million units before the pandemic and 16.3 million units during the pandemic. E-cigarette unit sales increased during the first half of 2019, peaking at approximately 15.2 million units in the period ending August 24, 2019 (Figure). This growth was followed by a steep decline during August, September, and October 2019, after which sales leveled off for the rest of 2019. Sales declined through early 2020, reaching the lowest unit sales of approximately 11.9 million units in the period ending February 8, 2020. E-cigarette unit sales jumped by about 1.6 million units during the 4-week period ending April 4, 2020 (model-based MPC = 19.4%; *P* < .001), and further increased with fluctuation, peaking at 17.2 million units for the period ending September 19, 2020. Unit sales then remained stable with a minor decline and fluctuation during the rest of the study period. Unit sales remained higher at the end of 2020 than they had been at the end of 2019 and were lower in June 2021 than in June 2020.

The 4-week e-cigarette unit sales increased on average by 37.1% (*P* < .001) during the pandemic compared with the prepandemic period (Table 1). The stockpiling period was associated with 25.6% (*P* < .001) greater 4-week unit sales.

### Cigar sales

The average 4-week unit sales of cigars were 204.1 million units before the pandemic and 222.6 million units during the pandemic (Figure). Cigar unit sales showed little fluctuation, from about 206.6 million units in the period ending April 6, 2019, to 202.2 million units in the period ending January 11, 2020. During the period from March 8 through May 30, 2020, cigar unit sales grew, peaking at 242.2 million units, followed by a sharp decline through the rest of 2020 and into January 2021. Unit sales then increased during the period ending April 3, 2021, and decreased again through June 2021. The pandemic was associated with an average of 26.1% (*P* < .001) greater unit sales compared with the prepandemic period. The stockpiling period was associated with 11.3% (*P* < .001) greater 4-week unit sales.

### NRT product sales

The average 4-week unit sales of NRT products before the pandemic was 315.1 million units and during the pandemic, 299.0 million units (Figure). NRT unit sales remained relatively steady until early 2020, ranging between 306.7 million and 322.8 million units per 4-week period (model-based MPC = 0.2%; *P* = .02). NRT unit sales then increased sharply, to about 336.0 million units, in the period ending April 4, 2020 (model-based MPC = 5.7%; *P* < .001); this was followed by a large drop to 278.9 million units in the next period ending May 2, 2020 (model-based MPC = −15.2%, *P* < .001). NRT unit sales increased slightly during May, June, and July 2020 but did not recover to prepandemic levels.

The 4-week NRT unit sales were lower, on average, by 13.1% (*P* < .001) during the pandemic compared with the prepandemic period. The stockpiling period was associated with 5.6% (*P* < .001) greater 4-week unit sales.

A sensitivity analysis considering the effect of Tobacco 21 and FDA’s regulation on flavored e-cigarettes sales indicated that the estimated associations of tobacco products and NRT unit sales with the pandemic period and stockpiling period were mostly similar to the adjusted association after controlling for these 2 policy events, which implied the main findings were robust (Table 2). The sensitivity analysis using the Prais–Winsten method to estimate model parameters indicated that the improvement in the autocorrelation function was negligible, and the change in the slope estimates was minimal, which suggested the robustness of the main findings. 

**Table 2:** Estimated Association of Unit Sales of Tobacco Products and Nicotine Replacement Therapies With COVID-19 Pandemic and Stockpiling After Adjusting for Policy Events, January 2019–June 2021^a^

Product	Estimated association with COVID-19 pandemic^b^		Estimated association with the period of potential stockpiling^c^	
	Estimate effect, % (95% CI)	*P* value^d^	Estimate effect, % (95% CI)	*P* value^d^
Cigarettes	8.6 (0.7 to 17.2)	.03	9.5 (2.5 to 17.0)	.01
E-cigarettes	30.6 (15.3 to 47.9)	<.001	19.6 (7.3 to 33.4)	.002
Cigars	24.1 (18.2 to 30.3)	<.001	9.5 (4.0 to 15.3)	.001
NRT products^e^	−14.4 (−18.0 to −10.6)	<.001	3.9 (0.1 to 7.9)	.04

a Policy events were 1) the raising of the minimum legal purchase age for tobacco products to 21 years (Tobacco 21) on December 20, 2019, and 2) the US Food and Drug Administration’s regulation on flavored e-cigarette sales on February 6, 2020. For cigarette and e-cigarette unit sales, linear and quadratic terms were included in the periods before the pandemic and another set of linear, quadratic, and cubic terms were added for the period during the pandemic. For cigar unit sales, linear and quadratic terms were included before and during the pandemic. For NRT unit sales, a linear term was added before the pandemic, and a set of linear, quadratic, and cubic terms were included during the pandemic.

b The pandemic was defined as beginning with the 4-week periods starting on April 5, 2020, through June 26, 2021. The prepandemic period was defined as the 4-week periods before April 5, 2020.

c The stockpiling period was defined as the 4-week period from March 8, 2020, through April 4, 2020.

d Determined by *t* tests; *P* ≤ .05 considered significant.

e NRT products sold over the counter; includes gum, patches, and lozenges.

## Discussion

Our study findings suggest that 4-week unit sales of cigarettes, e-cigarettes, and cigars increased and unit sales of NRT products decreased during the COVID-19 pandemic compared with the prepandemic period, which are in line with prior studies that separately examined sales of cigars, cigarettes, and NRT products (8–10). Although data from the National Health Interview Survey documented a decrease in the overall prevalence of tobacco use from 2019 to 2020 (21), our study’s findings may reflect longer-term stockpiling behaviors (ie, tobacco use went down, but sales went up) that were not captured by the prepandemic stockpiling period. National Health Interview Survey data also indicated a decline in the intensity of tobacco use, with adults generally smoking fewer cigarettes per day in 2020 than in 2005 (21). Therefore, our study’s findings may also shed light on more immediate changes in the intensity of tobacco use during the pandemic and complement prior studies that indicate that, because of stress caused by COVID-19 and the effects of social distancing and quarantine policies, the intensity of commercial tobacco use may have increased during the pandemic among people who smoked cigarettes or led to relapses among those who had previously quit (3,4,14).

Our study findings of substantial increases in 4-week unit sales of tobacco and NRT products from March 8 to April 4, 2020, suggest that people engaged in stockpiling behaviors in anticipation of or in response to pandemic restrictions. The model-estimated 12.5% increase in cigarette unit sales associated with the stockpiling period compared with all other 4-week average unit sales (697 million units) could equate to an increase of 87.1 million packs/units in total and 2.8 packs/units per adult who smoked cigarettes, with 30.8 million adults smoking cigarettes in 2020 (21). By the same token, the stockpiling period is associated with an approximate increase of 0.4 units per adult who used e-cigarettes and 2.8 units per adult who used cigars, with 9.1 million adults using e-cigarettes and 8.6 million using cigars in 2020 (21). The adjusted association controlling for Tobacco 21 policies and FDA’s regulation of flavored e-cigarettes that coincided with the stockpiling period was similar to the association without controlling for these policy events, suggesting that the sales increases were not confounded by these policy events. This finding is consistent with prior studies showing that stockpiling and bulk purchasing behaviors were common for consumers during the pandemic (8,11,12,22,23).

The pattern of unit sales of tobacco products and NRT products during the pandemic was different from the pattern during the prepandemic period. After April 4, 2020, the average 4-week unit sales compared with prepandemic periods remained substantially higher for e-cigarettes (16.3 million units vs 13.2 million units) and cigars (223.6 million vs 204.1 million units) and similar for cigarettes (692.0 million vs 701.2 million units). The pandemic was associated with increases in unit sales of cigarettes (11.5%), e-cigarettes (37.1%), and cigars (26.1%) compared with the prepandemic period. On the other hand, we observed possible stockpiling of NRT products during the 4-week period from March 8 through April 4, 2020, but the average 4-week unit sales of NRT remained overall substantially lower at 299.0 million units during the pandemic period compared with 315.1 million units during the prepandemic period. Our study found that the pandemic was associated with a 13.1% reduction of NRT unit sales compared with the prepandemic period. Stockpiled NRT products might have been consumed gradually, lowering additional purchases for the rest of 2020 through June 2021. Additionally, the increasing sales of cigarettes, e-cigarettes, and cigars and decreasing sales of NRT products during the pandemic period versus the prepandemic period may also have reflected relapse of former smokers and other tobacco users. During the pandemic, people may have lost jobs or income and perhaps their access to insurance that covers health care and medications, and they may have had limited access to the health care system that advises, prescribes, and counsels patients on how to use NRT products. Places that sell NRT products might have been less accessible because of closures or limited hours. In some states, Medicaid covers only prescription NRT products, so the decline in OTC NRT sales may signal a shift to increased prescription NRT products, which NielsenIQ data did not capture. These findings might suggest that over-purchase of tobacco products in the short term could lead to mid-term or long-term increases in purchases of tobacco products, which might be caused by the addictive nature of nicotine (13). Possibly for that reason, the stockpiling of NRT products did not lead to mid-term or long-term increases in purchases of NRT products during the pandemic, which might be because NRT products are less addictive than tobacco products and do not create a new addiction (24). Additionally, if people who used stockpiled NRTs to attempt to quit were successful, they might not have continued to use them throughout the pandemic.

Unit sales of e-cigarettes increased more and remained higher during the pandemic (vs prepandemic) compared with unit sales of cigarettes and cigars, while the unit sales of NRT products decreased during the pandemic. The outbreak of e-cigarette, or vaping, product use–associated lung injury (EVALI) that occurred from March 2019 to February 2020 (25) might have contributed to the decline in e-cigarette unit sales in the months before the pandemic (26). The higher percentage increase in e-cigarette unit sales compared with cigarettes and cigars might be due, in part, to a post-EVALI “bounce back” in sales near the start of the pandemic. Although prior studies reported an increasing number of quit attempts during the pandemic (4–6), it has been suggested that e-cigarettes are being used as a nicotine alternative by persons attempting to quit cigarette smoking (27). E-cigarettes can benefit adults who smoke and who are not pregnant if used as a complete substitute for regular cigarettes and other smoked tobacco products (28). However, e-cigarettes are not currently approved by FDA as a tobacco cessation product and the evidence is currently insufficient to recommend e-cigarettes for smoking cessation among adults, including pregnant adults (28). Additionally, e-cigarette liquids and aerosols contain hazardous and potentially hazardous additives and contaminants whose risks have yet to be fully explored (28). Increasing the accessibility and promotion of evidence-based tobacco cessation resources (ie, via increased access to cessation medications, virtual counseling, and quitlines for smoking cessation) remains important to help support and increase successful cessation, including during public health emergencies like the COVID-19 pandemic.

### Limitations and strengths

This study is subject to several limitations. First, NielsenIQ data do not include online retail sales or sales from tobacco specialty stores or vape shops. NielsenIQ data do not assess sales of all OTC NRT products or all prescribed cessation medications, resulting in an underestimation of sales of OTC NRT products and other cessation medications. This lack of complete information on tobacco and NRT sales can affect the study’s internal and external validity because the data may not adequately represent overall sales, thus introducing bias and limiting the generalizability of the findings. Second, because the sales data do not provide demographic information on buyers, we could not conduct subgroup analyses. Third, the start date of the COVID-19 pandemic could not be mapped exactly onto NielsenIQ data; these data are aggregated and provided in 4-week intervals only. Therefore, consistent with the literature (12), we defined the 4 weeks from March 8 to April 4, 2020, as the hypothetical stockpiling period. However, because a national emergency was declared on March 13, 2020, and the first stay-at-home order was issued in California on March 19, 2020 (earlier than April 4, 2020), the estimated effect of hypothetical stockpiling might partially result from a response to the pandemic. Therefore, the increase in unit sales of tobacco products during the pandemic compared with the prepandemic period could be larger than estimated, and the decrease in unit sales of NRT products during the pandemic compared with before the pandemic could be smaller than estimated. Because monthly aggregated data do not capture fluctuations within each month, they do not account for instant changes in sales within a month. Moreover, this national time series analysis did not capture state-by-state differences in the timing of stay-at-home orders and, thus, could have missed potential differential effects in states on sales. The COVID-19 pandemic is a unique event that could limit the generalizability of our study to other periods or contexts, and people who stockpiled tobacco products and NRT products before the pandemic may also differ systematically from those who did not. In addition, there is little way to know the influence of the supply chain during the pandemic, when there may have been weeks when product availability was limited. Finally, because our analysis was descriptive, causality cannot be inferred.

Notably, our study, which used retail scanner data, complements prior studies that used survey data on cigarette and e-cigarette use by comprehensively describing national trends in sales of tobacco products (cigarettes, cigars, e-cigarettes) and OTC NRT products. Previous studies used survey data that focused on cigarettes and e-cigarettes, but information on cigar and NRT products sales was limited; this study addressed those gaps.

### Conclusions

Our models found that 4-week unit sales of cigarettes, e-cigarettes, and cigars during the COVID-19 pandemic increased, compared with the prepandemic period, but 4-week unit sales of OTC NRT products decreased. Our study also suggests that stockpiling behavior occurred for all products in anticipation of or in response to the pandemic lockdown. The findings from our study have important implications. Increases in tobacco product sales during the pandemic could affect public health in 2 ways: first, increases could indicate increased intensity of tobacco use, which could affect both short-term and long-term health (29), and second, cigarette smoking is a risk factor for severe COVID-19 illness (2). Larger decreases in sales of NRT products during the COVID-19 pandemic may indicate a need for increased promotion of and access to FDA-approved cessation resources and messaging to support and motivate cessation attempts, particularly among people who had not been trying to quit. Consideration of context-tailored strategies during public health emergencies, such as increased promotion of cessation information and resources, could encourage people who use tobacco products to quit. For example, virtual smoking cession programs, such as online therapy sessions and peer support groups through social media platforms, could provide tailored strategies to cope with pandemic-related stress and anxiety. To help tobacco users access cessation resources, health care providers could also offer NRT products via mail or curbside pickup. Public health campaigns could shift their messaging to address the pandemic’s unique challenges, such as highlighting the benefits of quitting tobacco use. Such benefits include the improvement of lung health, the reduction in risk of severe COVID-19 complications, and financial benefits, particularly among people facing job loss or financial strain.
